# Development and application of chlorine-free antifreeze and dust suppression agent for transportation pavement of surface coal mines in Alpine areas

**DOI:** 10.1371/journal.pone.0329471

**Published:** 2025-09-17

**Authors:** Xiaoliang Zhao, Shaohui Feng, Fangwei Han, Bandna Bharti, Zhao Lian, Zhaolin Shen, Jing Du, Yide Li, Qi Shi

**Affiliations:** 1 Ordos Institute of Liaoning Technical University, Ordos, China; 2 Environmental Science and Engineering Department, Liaonning Technical University, Fuxin, Liaoning, China; 3 College of Safety Science and Engineering, Liaoning Technical University, Huludao, Liaoning, China; 4 Department of Chemistry, DAV University, Jalandhar, Punjab, India; Mirpur University of Science and Technology, PAKISTAN

## Abstract

Truck transportation pathways in surface coal mining operations are significant sources of dust emissions. Currently, water sprinkling and aqueous dust suppressants are commonly used on haul roads in surface coal mines. However, in the alpine regions of northern China, this practice poses serious safety risks due to road surface freezing. Addressing the conflict between effective dust suppression and the risk of icing on haul roads presents a significant practical challenge. To overcome this issue, a chlorine-free antifreeze dust suppressant was developed through a systematic process involving dust characterization, monomer screening, orthogonal formulation design, performance testing, and field validation. The investigation identified sodium dodecylbenzene sulfonate, glycerol, polyacrylamide complexed with ethylene glycol, potassium acetate, and potassium formate as the optimal monomer materials. The dust suppressant shows minimal corrosiveness, resistance to wind erosion and evaporation, and has a freezing point of – 35.3°C, representing a substantial improvement over traditional water and aqueous dust suppression methods. Field testing demonstrated that the formulated chlorine-free antifreeze dust suppressant extended effective dust suppression duration to 8 days. Moreover, the economic costs associated with its application were deemed low. These findings emphasized the efficacy and practical utility of the chlorine-free antifreeze dust suppressant for mitigating dust emissions along transportation routes within alpine open pit coal mines.

## 1. Introduction

Coal mining operations in China produce significant amounts of dust, particularly from the transportation roads within surface coal mines, which can constitute 70% to 90% of total dust emissions within mining areas [1–3]. On transportation roads in opencast coal mines, each truck generates approximately 2.5 to 4.8 kilograms of dust per kilometer, with PM_10_ particles accounting for more than 60% of the total and representing the most dominant and harmful fraction [4].This dust not only poses environmental challenges but also jeopardizes the health of workers, increasing the risks of pneumoconiosis and cancer [5–7]. Moreover, the fine particles of dust can infiltrate machinery, diminishing equipment performance and lifespan [8]. Studies have shown that during peak production periods, PM_10_ concentrations in some industrial areas can exceed the WHO’s annual average limits by two to three times. Dust generated from coal production and transportation can reduce local visibility by 15–20% and often contains heavy metals, posing serious health risks to workers and nearby residents. As a result, the increasing severity of dust pollution on workfaces and transportation routes has become a significant global public health concern [9].

Scholars worldwide have made substantial steps in developing dust suppressants to address road dust pollution. For instance, Liu investigated various materials such as bitter vegetable water dispersion, chitosan integrated polymer, rosin polyethylene glycol citrate, hydroxypropyl starch, calcium chloride, sodium dodecylbenzene sulfonate, and sodium tungstate to formulate dust suppressants, achieving an effective dust suppression period of 6 days with dust suppression rates ranging from 83.2% to 87.2% for PM_10_ and PM_2.5_ [10]. However, Liu synthesized a chemical composite dust collector with calcium chloride (CaCl_2_) and magnesium chloride (MgCl_2_), which exhibited strong moisture absorption capabilities to prevent dust initiation under mines [11]. Yet, the corrosive nature of inorganic salt containing moisture absorbent dust suppressants poses risks to site infrastructure and equipment components. Alternatively, Weatherman explored oils and fats such as lavender oil, glycerin, and pine oil in combination with lignosulfonate, sodium dodecyl sulfate (SDS), and sodium borate as surfactants to design a climate specific dust suppressant suitable for spraying [12]. Additionally, Yu developed a dust suppressant using mussel adhesion proteins rich in phenolic hydroxyl and lysine residues, demonstrating good adhesion and consolidation properties [13]. The incorporation of organic phases into aqueous solvents effectively lowered the freezing point (approximately −15.3°C) of the solution, imparting ice precention and water retention characteristics. These studies highlight the urgent need for better ways to deal with dust in coal mines. Researchers are trying out different materials and mixtures to make the air cleaner and safer for both the environment and people’s health [14–17]. Currently, chlorine-based inorganic salts or organic polymers are commonly used for dust suppression, with limited exploration of chlorine-free antifreeze and dust suppressants for controlling dust on transportation roads in alpine surface coal mines. Over time, chlorine containing suppressants can corrode truck tires in mining areas, reducing their lifespan [18–20]. Research has revealed that rubber exposed to 5% sodium chloride solution for 90 days experiences a 15–20% decline in tensile strength. Inhibitors based on hypochlorite cause visible cracks on truck tire surfaces within 30 days [21].The research aimed to develop a dust suppressant specifically tailored to effectively control dust on transportation roads in alpine surface coal mines. Through screening and orthogonal experiments, we identified a formulation capable of addressing existing challenges such as limited effectiveness and application difficulties. The urgent need for a highly efficient, chlorine-free, user-friendly, environmentally sustainable, and freeze resistance dust suppressant is evident.

## 2. Experimental materials and methods

### 2.1. Collection and preparation of road dust samples

The geographical coordinates of this sampling site are shown in Table 1. Dust samples were collected from the truck haul roads of the North Hollinhe surface coal mine in Tongliao, Inner Mongolia (119°32′–119°45′E, 45°28′–45°42′N), a region facing severe dust pollution due to widespread surface mining activities. Field tests were conducted to evaluate suppressant effectiveness under high particulate concentrations. The area’s cold winters (average temperatures between –25°C and –30°C) also provided a rigorous setting to test the low temperature performance of chlorine-free antifreeze suppressants, supplying key data to address dust control challenges in northern China. The raw samples collected were first primary crushed using a jaw crusher. Then the samples were ground by a roller ball mill for 9 hours. Subsequently, the dust samples on the ground were vacuum dried at 25°C until a constant weight was reached to obtain experimental dust samples for subsequent analysis. Sampling efforts targeted the surface coal mine haulage pavement, totaling eight sampling points. The opposite lanes of Road 4 and 5 (each approximately 500 meters in length) were selected as sampling sections, and dust samples were collected from the surface layer (approximately 10 centimeters) at each sampling point (spaced about 50 meters apart). Chemical analysis of the dust present on Truck haulage road surface in open pit coal mines was conducted using the XRF-1800 X-ray fluorescence spectrometer, with the outcomes outlined in Table 2.

**Table 1:** Map of sampling sites and test geographic locations.

Sampling point	Latitude and longitude	Distance(m)	Sampling route
1	119°39.000′E, 45°58.000′N	0	4
2	119°39.039′E, 45°58.000′N	50	
3	119°39.077′E, 45°58.000′N	100	
4	119°39.116′E, 45°58.000′N	150	
5	119°39.000′E, 45°58.027′N	0	5
6	119°39.039′E, 45°58.027′N	50	
7	119°39.077′E, 45°58.027′N	100	
8	119°39.116′E, 45°58.027′N	150	

**Table 2:** Chemical composition content of dust on transport road surface of opencast coal mine.

Ingredient	SiO_2_	Fe_2_O_3_	Al_2_O_3_	K_2_O	CaO	Na_2_O	TiO_2_	MnO
quantity contained (%)	64.138	13.539	13.313	3.782	3.091	1.168	0.898	0.067

According to the test results, the chemical components in the transportation road dust are mainly SiO_2_, Fe_2_O_3_, Al_2_O_3_, followed by K_2_O, CaO, Na_2_O, and containing a small amount of TiO_2_ and MnO. The content of SiO_2_ is as high as 64.138%, which indicates that this kind of dust is difficult to dissolve in water, and the dust with high content of Si element is the most hazardous dust for pneumoconiosis (silicosis). Poor water solubility limits the effectiveness of conventional dust suppression methods. Sprinkling water alone is insufficient to achieve satisfactory dust control, so chemical reagents must be used to convert the dust’s hydrophobic properties into hydrophilic ones, ultimately enhancing dust suppression efficiency [22–24].

### 2.2. FT-IR analysis of road dust

**Fig 1:**
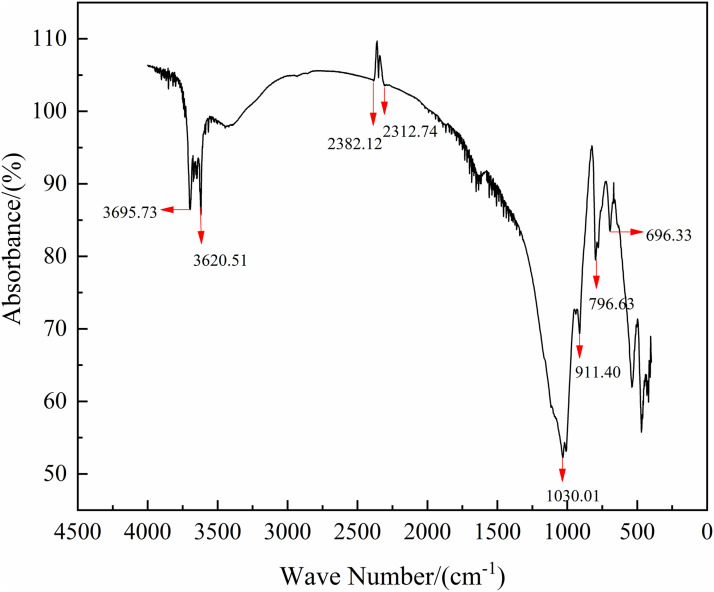
The FTIR spectra of the samples of road dust.

To investigate the surface coal mine transportation road dust belongs to the hydrophilic or hydrophobic system [25,26], this experiment uses Fourier infrared spectrometer to measure the dust sample, and the absorption peaks of infrared spectra are used to deduce the characteristic groups of the dust, and then determine whether the dust is hydrophilic or not [27]. The Fourier infrared spectrometer is shown in the Fig 1.

The data plots reveal strong absorption bands around 1030.01 cm^-1^ and 911.40 cm^-1^, corresponding to Si-O-Si anti-symmetric stretching vibrations, while absorption peaks at 796.63 cm^-1^ and 696.33 cm^-1^ represent symmetric stretching vibrations of Si-O. These observations indicate the presence of silicon oxides in the dust samples. Furthermore, higher proportions of silicon oxides contribute to decreased affinity between the dust particles and water. The experimental dust exhibits hydrophobic characteristics due to its elevated SiO_2_ content.

### 2.3. Monomer material screening experiments

By reviewing the relevant information, the following materials were selected for monomer screening experiments: sodium dodecylbenzene sulfonate (SDBS), cocoamidopropyl betaine (CAB), SDS, cetyltrimethylammonium bromide (CTAB) were selected as surfactants; Polyacrylamide (PAM), carboxymethylcellulose (CMC), carboxymethylcellulose sodium (CMC-Na), soluble starch (SLS) were used for binders; Sodium polyacrylate (PAAS), triethanolamine (TEA), glycerol (GLY) were selected as moisturizers; Ethylene glycol (EG), Potassium formate (PF), Potassium acetate (PAC) were selected as antifreezes. For each performance index, three replicate tests were conducted.

#### 2.3.1. Surfactant monomer experiment.

**Fig 2:**
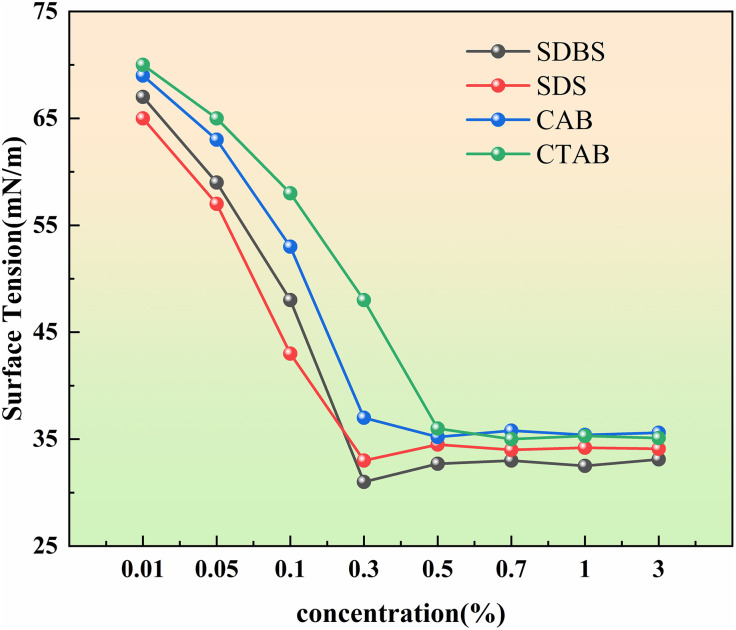
The surface tension tendency of surfactants in different concentrations.

Surfactants have the capability to modify the surface tension of a solution [28,29]. Lower surface tension facilitates the spreading of liquid on solid surfaces, enhancing its penetration and wetting properties, which is essential for antifreeze applications [30]. Prior to establishing the experimental parameters for surfactants, solutions having different mass concentrations which range from 0.01% to 3.00% were made ready and stored as backups on the experimental bench. The surface tension of these selected reagents was then measured using the JK99C fully automated tensiometer employing the method of the platinum plate. Fig 2 illustrates the surface tension values of different surfactants.

The surface tension of SDBS and SDS reached the minimum values of 31.02 mN/m and 33.14 mN/m, respectively, at approximately 0.30% mass concentration. Similarly, CAB and CTAB exhibited minimum surface tension values of 35.2 mN/m and 35.2 mN/m, respectively, within the mass concentration range of 0.50% to 0.07%. Thus, SDBS at a mass concentration of 0.30% was deemed more effective in reducing surface tension [31,32].

#### 2.3.2. Binder agent monomer experiment.

Viscosity serves as a crucial performance indicator for assessing the bonding strength of the binder and the ease of spraying [33–35]. In accordance with the methods outlined in GB/T10247-2008 Viscosity Measurement Methods,“viscosity measurements were conducted using an NDJ-8S viscometer (with No. 1 rotor, operating at 60 r/min). This made it possible to determine the viscosity of the solution and that of water at different temperatures, facilitating a comparative analysis of viscosity characteristics [36]. To determine viscosity, polyacrylamide, carboxymethylcellulose, sodium carboxymethylcellulose, and soluble starch were prepared in mass concentrations ranging from 0.01% to 0.50%. These solutions were thoroughly dissolved and utilized for experimental viscosity assessments.

**Fig 3:**
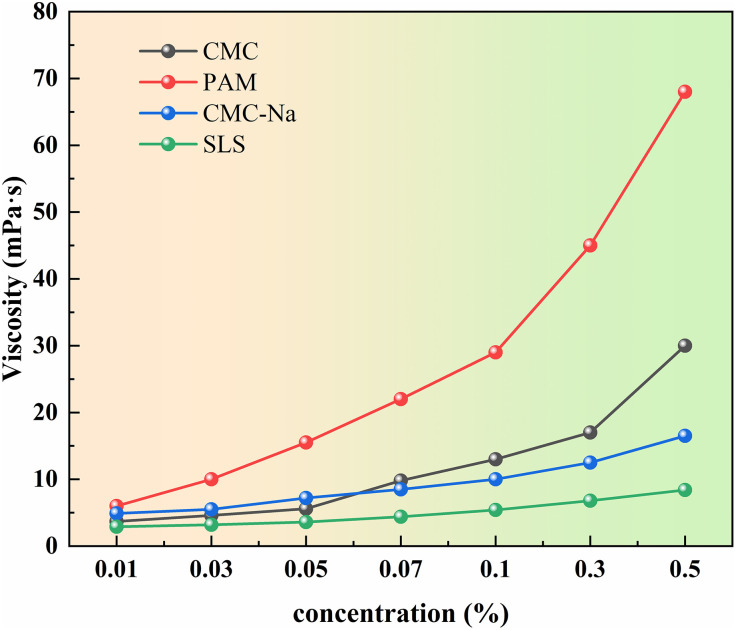
Changes of binder viscosity at different concentrations.

Fig 3 illustrates the results of viscosity measurements for different binders. As concentration increases, viscosity rises for all four binders. Particularly, the viscosity of polyacrylamide solution escalates steeply with increasing concentration, from 6.0 mPa·s at 0.01% to 68.0 mPa·s at 0.5% mass concentration. Among the tested concentrations, the bonding effects of the binders follow this order: polyacrylamide demonstrates the strongest bonding effect, followed by carboxymethyl cellulose, while sodium carboxymethyl cellulose and soluble starch exhibit the weakest bonding effects. Considering practical application, excessively high viscosity can complicate spraying and reduce dust suppressant penetration. Hence, it’s recommended to utilize a mass concentration of around 0.5% for polyacrylamide in subsequent experiments to strike a balance between viscosity and performance.

#### 2.3.3. Humectant monomer experiment.

**Fig 4:**
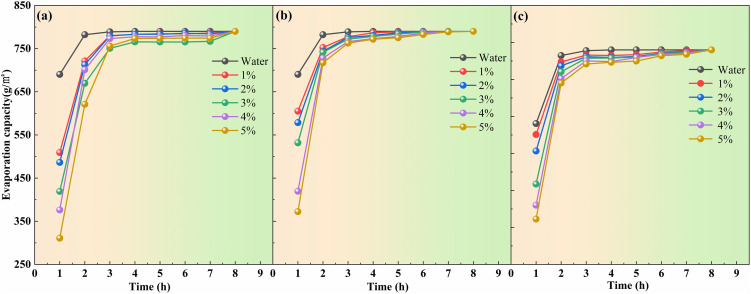
Evaporation capacity of Propylene glycol solution, Sodium polyacrylate, and Triethanolamine solution.

Humectants play a crucial role in mitigating dust dispersion by forming a moisture film on dust surfaces, thereby reducing airborne spread. Additionally, they contribute significantly to inhibiting dust emission from its sources [37,38]. To assess the moisturizing efficacy, solutions of sodium polyacrylate, triethanolamine, and propylene glycol were prepared at varying concentrations (1.0%, 2.0%, 3.0%, 4.0%, 5.0%, and 6.0% by volume). These solutions underwent evapotranspiration resistance testing to determine their effectiveness. Statistical analysis of evaporation data was conducted to inform the selection of moisturizing agents for the current composite dust suppressant formulation, as depicted in Fig 4.

According to the graph, the evaporation of the three humectants increases with time, displaying varying degrees of growth. A notable trend was that higher evaporation corresponds to weaker moisturizing effects, with evaporation eventually reaching a plateau where significant changes cease. Following 1 hour of blast drying, the evaporation ranking of the three humectants was as follows: propylene glycol > sodium polyacrylate > triethanolamine. Notably, after this period, the mass concentration of 5% yields the minimum evaporation, while 1% yields the maximum for all three humectants. After 2 hours of blast drying, the evaporation trends remain consistent with the first hour. In the time span from the third to the eighth hours of blast drying, the evaporation for all three undergoes stabilization and attains maximal magnitudes. Consequently, glycerol demonstrated superior moisturizing performance, consistently yielding the lowest evaporation. It was recommended to incorporate approximately 3% mass concentration of glycerol for subsequent orthogonal experiments on dust suppressants.

#### 2.3.4. Antifreeze components compounding efficiency experiments.

Antifreeze serves as a critical measure to prevent road surface icing, particularly in low temperature environments, thereby averting safety hazards such as truck tire skidding [39,40]. During on-site testing, the ambient temperature dropped as low as −30°C, requiring the dust suppressant to maintain a freezing point below this threshold to ensure effective application. The synergistic outcomes of antifreeze experiments are summarized in Table 3. Comparing the 1st and 2nd groups, it’s evident that the use of ethylene glycol monomer results in a measured freezing point of −33.7 °C. Upon adding 10% acetic acid methyl, the freezing point decreases to −34.8 °C, indicating that potassium acetate can enhance ethylene glycol’s ability to lower the freezing point, show casing a synergistic effect between inorganic salt and alcohol depressants. However, this combination may influence the solution’s acidity and alkalinity. Groups 2, 3, and 4 demonstrate that potassium formate also contributes to reducing the freezing point in compound solutions. Further analysis from groups 4–6 confirms that the synergistic effect of the two inorganic salts is more pronounced. Notably, the freezing point increases from −34.9°C to −35.3°C from group 5 to group 6, indicating potassium formate’s superior synergistic effect over potassium acetate. To ensure optimal freezing point reduction at low concentrations, experiments in groups 7, 8, and 9 were conducted. While the freezing point values of these groups exceed −35°C, comparative analysis with Groups 7–9 showed that the Group 6 not only offered a clear cost advantages but also achieved optimal environmental safety and performance through a scientifically optimized ethylene glycol concentration. Considering field requirements along with freezing point, pH value, cost, and concentration, it’s suggested to adopt the compound solution from the 6th group as a more effective antifreeze and coagulation reduction.

**Table 3:** Determination of synergistic effect of three kinds of antifreeze material compounding.

Serial Number	Mass fraction (chemistry)/%				Freezing point (°C)
	Ethylene glycol	Potassium acetate	Potassium formate	Water	
1	50	0	0	50	−33.7
2	40	10	0	50	−34.8
3	35	10	5	50	−35.4
4	30	10	10	50	−36.4
5	30	10	5	55	−34.9
6	30	5	10	55	−35.3
7	25	10	10	55	−36.1
8	25	10	8	59	−35.7
9	25	8	10	57	−35.9

### 2.4. Orthogonal experimental design

To ensure optimal dust suppression performance, four functional materials were chosen based on previous monomer material screening experiments: humectant glycerol (H), binder polyacrylamide (Q), surfactant sodium dodecylbenzenesulphonate (A), and antifreeze such as ethylene glycol, potassium acetate, potassium formate (T). An orthogonal table with four factors and three levels was designed for experimentation, employing the polar deviation method for analysis. The aim was to determine the ideal mass concentration of each material and formulate the optimal dust suppressant [41]. The orthogonal experimental factor level table is presented in Table 4.

**Table 4:** Orthogonal test factor level.

Serial Number	(H)	(Q)	(A)	(T)
1	2.50%	0.05%	0.25%	31% + 4% + 9%
2	3%	0.06%	0.35%	30% + 5% + 10%
3	3.50%	0.07%	0.45%	29% + 4% + 11%

The impact of each factor on the effectiveness of the dust suppressant is evaluated, four factors at three different levels were chosen based on practical requirements. Orthogonal experiments were conducted using the L9 (3^4^) orthogonal table. In the laboratory, simulated conditions were employed to measure the dust suppressant performance, focusing on viscosity, rate of penetration, resistance to evaporation, and the freezing point.

## 3. Results and discussions of orthogonal experiment

### 3.1. Results and analysis of viscosity measurement experiments

The relationship between viscosity values and bonding performance was observed, where higher values indicated better bonding [42,43]. This was reflected in the corresponding K values. Visually analyzing the data from Table 5 revealed that each formula solution achieved a maximum viscosity value of 25.5 mPa·s and a minimum of 16.5 mPa·s. Notably, as shown in Table 6, formulations H_3_, Q_3_, A_1_, and T_3_ were identified as optimal levels for factors H, Q, A, and T, respectively. Thus, the optimal formula was determined to be H_3_Q_3_A_1_T_3_. It was evident that Q has the most substantial effect on dust penetration rate, with the order of significance being R_Q_ > R_T_ > R_H_ > R_A_. Indicating that higher viscosity values enhanced the coagulation effect, facilitating the settling of dust particles. Conversely, lower viscosity values were associated with less effective dust suppression due to inadequate particle coalescence.

**Table 5:** Analysis of the results of the four influencing factors.

Serial Number	Viscosity (mPa·s)	Permeation Rate (cm/min)	Evaporation capacity (g/m^2^)	Freezing Point (°C)
1	16.5	0.31	116.69	−34.8
2	20.3	0.42	140.96	−35.1
3	25.4	0.52	90.1	−35.3
4	17.4	0.41	115.08	−35.4
5	20.2	0.58	159.26	−34.9
6	25.5	0.35	213.3	−35.2
7	17.1	0.54	93.81	−35.3
8	21.1	0.31	110.23	−35.5
9	25.3	0.43	147.08	−35

**Table 6:** Results of orthogonal experiments.

Factors		K1	K2	K3	R	The optimal formula
Viscosity(mPa·s)	H%	62.2	63.1	63.5	1.3	H_3_Q_3_A_1_T_3_
	Q%	51	61.6	76.2	25.2	
	A%	63.1	63	62.7	0.4	
	T%	62	62.9	63.9	1.9	
Permeation Rate (cm/min)	H%	1.25	1.34	1.27	0.09	H_2_Q_2_A_3_T_1_
	Q%	1.26	1.31	1.3	0.05	
	A%	0.97	1.26	1.47	0.5	
	T%	1.32	1.31	1.24	0.08	
Evaporation capacity (g/m^2^)	H%	347.75	487.64	351.12	139.89	H_2_Q_3_A_1_T_2_
	Q%	332.58	410.45	450.48	117.9	
	A%	440.22	403.12	343.17	97.05	
	T%	423.03	448.07	3415.41	132.66	
Freezing Point(°C)	H%	−105.2	−105.6	−105.8	0.6	H_3_Q_1_A_1_T_3_
	Q%	−105.5	−105.2	−105.5	0.3	
	A%	−105.5	−105.5	−105.5	0	
	T%	−104.7	−105.6	−106.2	1.5	

### 3.2. Results and analysis of permeation rate measurement experiments

The relationship between permeability values and performance was examined, with larger values indicating better permeability. Analyzing the data from Table 5 revealed that each formulation solution achieved a maximum penetration rate of 0.58 cm/min and a minimum of 0.31 cm/min. Notably, as shown in Table 6, formulations H_2_, Q_2_, A_3_, and T_1_ were identified as optimal levels for factors H, Q, A, and T, respectively. It was evident that A has the most substantial effect on dust penetration rate, with the order of significance being R_A_ > R_H_ > R_T_ > R_Q_. Thus, the optimal formulation was determined to be H_2_Q_2_A_3_T_1_, indicating that faster penetration rates enhanced the wetting performance, resulting in shorter dust wetting times and rapid inhibition of dust.

### 3.3. Results and analysis of evaporation resistance experiments

The relationship between evaporation and evaporation resistance performance was examined, with smaller values indicating better performance [44]. Upon analyzing the data from Table 5, it was observed that each formula solution achieved a minimum evaporation of 90.10 g/m^2^ and a maximum of 213.30 g/m^2^. Notably, as shown in Table 6, formulations H_2_, Q_3_, A_1_, and T_2_ were identified as optimal levels for factors H, Q, A, and T, respectively. Consequently, the optimal formula was determined to be H_2_Q_3_A_1_T_2_, indicating that lower evaporation rates enhanced water retention performance. It was evident that H has the most substantial effect on dust penetration rate, with the order of significance being R_H _> R_T_ > R_Q_ > R_A_. This resulted in prolonged moisture content on the dust, weakening its tendency for initiation and thereby improving dust suppression effectiveness.

### 3.4. Results and analysis of freezing point determination experiments

The freezing point value served as an indicator of antifreeze performance, with smaller values indicating better effectiveness. The freezing point value also reflects the likelihood of road surface icing conditions during winter spraying of the formula solution. A lower freezing point enhanced the antifreezing effect, improving the safety coefficient for road truck transportation and enhancing antiskid effects. It ensured the effective winter dust suppression while minimizing the risk of road icing. Analyzing the data in Table 5 revealed a minimum freezing point value of −35.50°C and a maximum of −34.80°C. As shown Table 6, optimal levels for factors H, Q, A, and T were identified as H_3_, Q_1_, A_1_, and T_3_, respectively, indicating that the best formula is H_3_Q_1_A_1_T_3_.

### 3.5. Determination of optimal formulation of dust suppressant

After conducting a four-factor three-level orthogonal test and performing polar analysis on viscosity, penetration rate, evaporation resistance, freezing point, and other indicators, the primary order of influence of different factors on each experimental group of dust suppressant formulations was determined. This analysis also identified the significant impact of each factor and identified the optimal formula.

Based on the analysis presented in Table 6, it was evident that factor H significantly influenced antievaporation, viscosity, and freezing point. Opting for H_2_ as the optimal level of factor H ensured better moisturizing effects, maintaining continuous moisture content in dust particles. Factor Q played a crucial role in viscosity and antievaporation, particularly impacting viscosity index formulation at level Q_3_, making it the optimal level for factor Q. Factor A primarily affected the penetration rate, showcasing the surfactant’s strong wetting ability in facilitating dust suppressant infiltration into hydrophobic dust. For viscosity, antievaporation, and freezing point, A_1_ was determined as the most optimal level for factor A. Factor T exhibited significant influence on viscosity, resistance to evaporation, and the freezing point, particularly making the greatest contribution to freezing point enhancement. This greatly improved the dust suppressant’s resistance to harsh winter temperatures, while also enhancing hygroscopicity and moisturizing properties. Factor T did not notably affect the penetration rate. Opting for T_3_ as the optimal level ensured optimal viscosity and freezing point indices. In summary, optimal formulation of the chlorine-free antifreeze dust suppressant was determined as H_2_Q_3_A_1_T_3_(sodium dodecylbenzene sulfonate 0.25%, glycerol 3%, polyacrylamide 0.07%, ethylene glycol 29%, potassium acetate 4%, and potassium formate 11%). Based on the optimal formulation of the orthogonal test, the evaporation resistance, viscosity, penetration rate and freezing point were determined, and the following were obtained: evaporation of 213.7 g/m^2^, viscosity of 25.7 mPa·s, penetration rate of 0.38 cm/min, and freezing point of −35.3°C.

## 4. Chlorine-free antifreeze dust suppressant property and performance test

### 4.1. Chlorine-free antifreeze dust suppressant property test

#### 4.1.1. Contact angle test.

**Fig 5:**
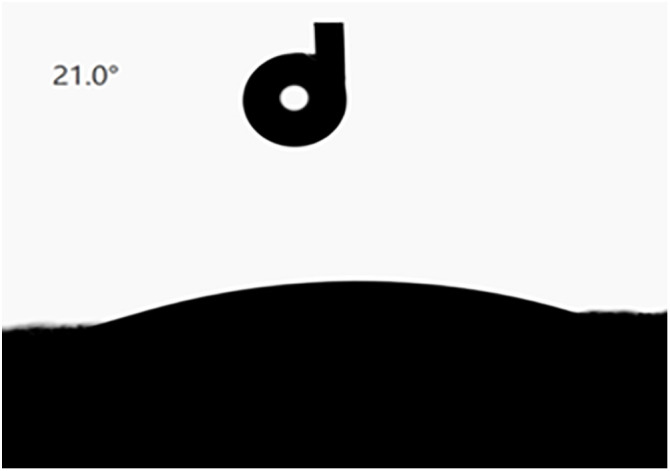
Contact angle of dust wetting by dust suppressant solution.

The wettability of the surfactant was detected by measuring its contact angle with the SCI3000 contact angle meter. For performance indexes, three replicate tests were conducted and the average values were calculated. After spraying dust suppressant or water dust suppression on the transportation surface coal mine, the size of the intermolecular force can be measured by the wetting contact angle θ. The larger the value of θ, the less significant the wettability of the solution to the dust [45]; on the contrary, it indicates that the wettability of the solution with regard to the dust is significant. The results of determining the contact angle of chlorine-free antifreeze dust suppressant wetting dust were shown in Fig 5.

Based on the contact angle measurement results, it was observed that upon dropping the dust suppressant solution onto the tablet, the contact angle was 21.0 °, while distilled water exhibited a contact angle of 83.4 ° on the dust. As the contact angle increases, the wetting effect of the solution on the dust worsens. Therefore, compared with distilled water, the suppressant solution demonstrated better wettability on dust, improving its ability to reduce roadway dust contamination during transport.

#### 4.1.2. Evaporation resistance test.

The evaporation rate was used as an index to test the antievaporation performance of dust suppressant solution and distilled water under high temperature conditions. Weigh 40 g of dust samples in a petri dish, take 10 mL of dust suppressant solution and distilled water were evenly sprayed on the surface of the dust, and then dried in a blower drying oven at 60 °C, weighed every 30 min until the constant weight.

**Fig 6:**
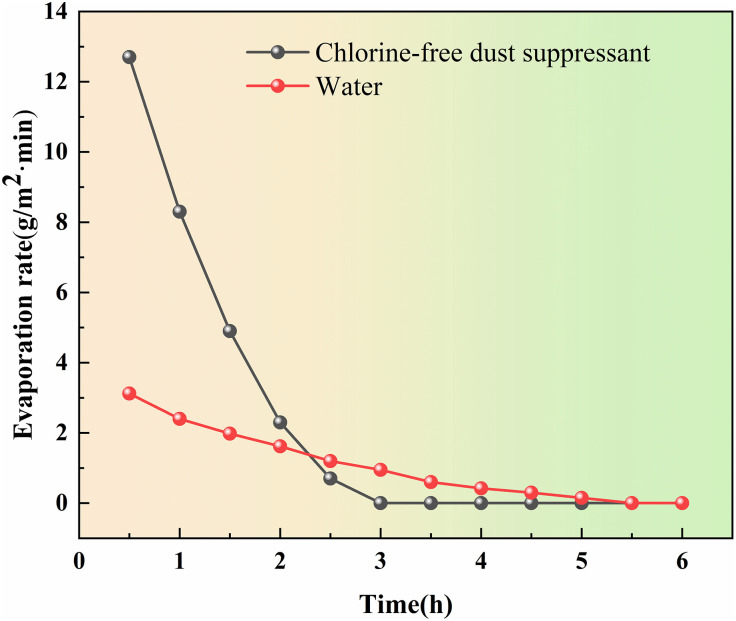
Evaporation rate variations.

Based on the data, Fig 6 depicted that over time, the water evaporation rate of dust samples sprayed with dust suppressant solution and distilled water gradually decreased. Eventually, it reached 0 at 3 hours and 5.5 hours, respectively. After the water evaporation of the distilled water-treated dust samples, the surface appeared relatively loose, without any film forming bonding phenomenon. On the other hand, dust samples treated with dust suppressant not only evaporated slowly but also exhibited solidification phenomenon on the surface after complete water evaporation. This indicates that the dust suppressant possessed a certain degree of evaporation resistance and adhesion.

#### 4.1.3. Viscosity test.

The suppressant exhibited optimal flow properties and dust control performance when viscosity ranges between 10–30 mPa·s. As shown in Table 7, higher temperatures correspond to lower viscosity values due to increased molecular motion and reduced intermolecular cohesion.

**Table 7:** The viscosity of the chlorine-free dust suppressant.

Temperature (°C)	5	10	15	20	25	30
Viscosity (mPa·s)	28.9	28.3	27.7	27.1	25.75	24.4

### 4.2. Chlorine-free antifreeze dust suppressant performance test

#### 4.2.1. Corrosion rate test.

In the corrosion experiments, common rubber, and carbon steel (16Mn) were immersed in both a chloride-free dust suppressant solution, distilled water and chlorine dust suppressant for 48 hours at room temperature (25°C). Rubber (XJ) and carbon steel (TG) specimens immersed in the chloride-free dust suppressant solution were labeled XJ1–3, TG1–3, while those immersed in distilled water were labeled XJ4–6, TG4–6. Rubber and carbon steel specimens immersed in the chloride dust suppressant solution were labeled XJ6–9, TG6–9. The weights of the specimens were recorded before and after immersion to determine the corrosion rate of the dust suppressant on rubber and carbon steel. The results are presented in Table 8.

**Table 8:** The corrosion rates comparison of rubber and carbon steel between dust suppressants.

	Number	surface area/cm^2^	Weight before experiment/ g	Weight after experiment/ g	corrosion rate/g·m^-2^·h^-1^	Average corrosion rate/g·m^-2^·h^-1^
Chlorine-free dust suppressant	XJ1	30.15	7.5123	7.5475	0.2432	0.2328
	XJ2	30.1	7.5025	7.5365	0.2353	
	XJ3	29.85	7.4201	7.4516	0.2198	
Water	XJ4	30.25	7.5213	7.5571	0.2466	0.2338
	XJ5	30.05	7.4995	7.5331	0.2329	
	XJ6	29.95	7.4876	7.5195	0.2219	
Chlorine dust suppressant	XJ7	30.05	7.52	7.5104	0.32083	0.31759
	XJ8	30.1	7.5018	7.5107	0.30069	
	XJ9	30.17	7.5312	7.5201	0.33125	
Chlorine-free dust suppressant	TG1	28.23	21.1052	21.0957	0.089	0.1001
	TG2	28.52	22.3862	22.3721	0.103	
	TG3	28.65	22.8148	22.7999	0.1083	
Water	TG4	28.71	22.9232	22.9106	0.0914	0.0885
	TG5	28.63	22.4008	22.3887	0.088	
	TG6	28.54	22.4107	22.3989	0.0861	
Chlorine dust suppressant	TG7	28.51	22.5019	22.4917	0.28958	0.3137
	TG8	28.62	22.5526	22.5417	0.28958	
	TG9	28.41	22.4826	22.4816	0.36181	

Under room temperature conditions of 25°C, the maximum corrosion rate of vulcanized rubber lanyards immersed in chloride-free dust suppressant solution and distilled water and chloride dust suppressant for 48 hours were 0.2432 g/(m^2^·h), 0.2466 g/(m^2^·h) and 0.331 g/(m^2^·h), respectively. The average corrosion rates were calculated as 0.2328 g/(m^2^·h), 0.2338 g/(m^2^·h) and 0.3176 g/(m^2^·h), respectively. Similarly, for carbon steel lanyards, the maximum corrosion rates under the same conditions were 0.1083 g/(m^2^·h), 0.0914 g/(m^2^·h) and 0.3618 g/(m^2^·h), with average corrosion rates of 0.1001 g/(m^2^·h), 0.0885 g/(m^2^·h) and 0.3137 g/(m^2^·h). The corrosion rates of rubber and carbon steel immersed in chloride-free dust suppressant solutions and distilled water do not differ significantly, but the corrosion rate of immersion in chlorinated dust suppressants exceeds that of chloride-free dust suppressant solutions and distilled water. It was evident that the chloride-free dust suppressant solution exhibited no significant corrosion effect on truck tires, metal parts of the frame, and other materials in surface mining areas. Therefore, under the conditions of antifreezing and dust suppression, it can be used without concern.

#### 4.2.2. The test for wind erosion resistance.

Resistance of wind erosion, i.e., the ability of sprayed effective liquid or untreated soil surface layer to resist windy weather, is can be can be considered as a performance indicator of the dust suppression capacity of the dust suppressant [46]. Using a blower and a portable anemometer to simulate the dust suppression effect of the dust suppressant sprayed road surface under wind force of 3 ~ 7, the erosion due to the wind of the samples under different wind force was calculated according to the formula of wind erosion rate.


$$\begin{array}{c} \text{\textbackslash{} \textbackslash{} }E=\frac{M-m}{M}\times 100\% \end{array}$$

E——The rate of wind erosion, %; M—— Weight before wind erosion, g; m——Weight after wind erosion, g.

**Fig 7:**
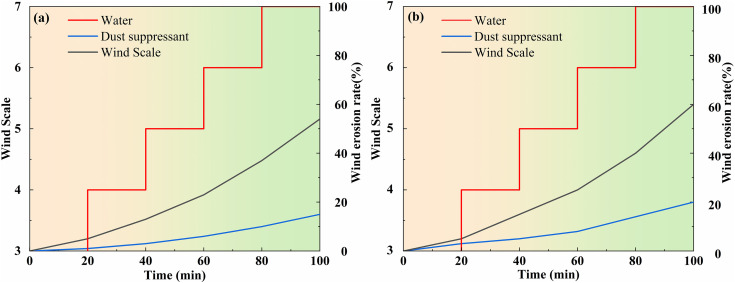
(a) and (b) represent the changes of wind erosion rate before and after milling under different wind levels, respectively.

Four glass plates were selected, cleaned, and dried before being weighed and recorded. Each glass plate, measuring 40 cm × 40 cm, was naturally homogenized with 500 g of dried dust samples. Two measures of uniform spraying were implemented: one that has a dust suppressant solution and the other with tap water, each applied at a rate of 2 L/m^2^. The experiment involved four glass plates: Glass plates 1 and 2 received no treatment, tap water spraying, and dust suppressant solution spraying. Glass plates 3 and 4 underwent the same treatment as 1 and 2 but required additional crushing using a rubber hose back and forth for 30 times to simulate the on-site truck transportation environment on mine road surfaces. Following treatment, all glass plates were placed in a 40°C oven for 30 minutes, then weighed. Subsequently, wind erosion was induced at wind levels ranging from 3 to 7 for 20 minutes each, with weights recorded after each erosion session. The erosion effects were observed upon completion. Finally, statistical data analysis was conducted on the rate of wind erosion of dust samples under each wind level or the rate of mass loss over time, along with the effectiveness of dust suppression, as depicted in Fig 7.

Based on the data presented in Fig 7, it was observed that the rate of wind erosion of the dust samples that have been sprayed with water and dust suppressant increased with the rise in wind level. Additionally, the wind erosion rate of dust samples after milling exceeded that before milling in both dust suppression methods. Specifically, the wind erosion rate of the dust samples which were sprayed with water was greater than that of the dust samples which were sprayed with the dust suppressant. Before milling, the wind erosion rates were 54% and 15% for spraying of water and dust suppressant, respectively, resulting in a difference of 29%. After milling, these rates increased to 60% and 20%, respectively, with a difference of 30%. This trend suggests that milling contributes to increased dust concentration. However, dust samples sprayed with dust suppressant exhibited lower wind erosion rates due to the presence of bonding material in the suppressant, which solidified the surface dust. Consequently, the dust suppressant demonstrated superior bonding and dust suppression properties.

#### 4.2.3. Toxicological testing of chlorine-free dust suppressant.

The chlorine-free dust suppressant, composed entirely of organic ingredients with a neutral pH of 7.5, maintained excellent antifreeze performance even at –40°C and exhibited no measurable corrosion. Toxicological assessments confirmed that all tested parameters, including heavy metal (Hg < 0.01 mg/kg, Cd < 4 mg/kg, Pb < 10 mg/kg, Cr < 4 mg/kg, As<0.06 mg/kg) contents and phytotoxicity, complied with national environmental and safety standards.

### 4.3. Analysis of on-site field tests

#### 4.3.1. Spray volume and dust suppressants routing.

**Fig 8:**
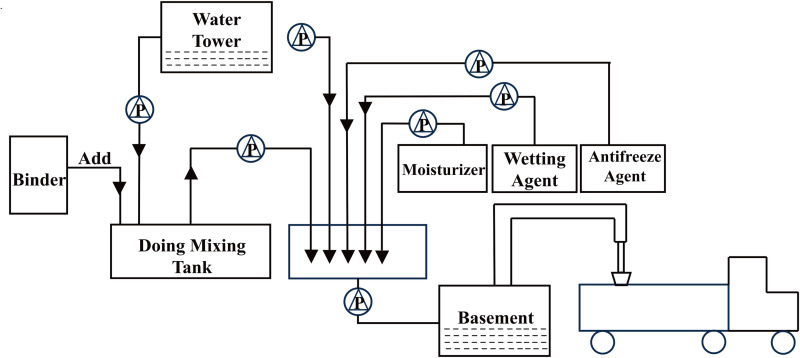
Flow chart of preparation, storage and loading of chlorine free antifreeze dust inhibitor solution.

The amount of dust suppressant sprayed on the opencast coal mine road significantly influenced the length of dust suppression, antifreezing performance, and spraying uniformity. Higher spraying volume facilitated spray evenly, extending dust suppression time and enhancing antifreezing effect, but increased operational costs and may impede truck movement. Conversely, lower volume lead to uneven spray, reducing both dust suppression and antifreezing efficiency, thus compromising protection. To optimize the spraying amount, dust suppressant solutions were prepared at dilution ratios corresponding to 0.8, 1.2, 1.6, 2.0, and 2.4 L/m². These were uniformly applied to petri dishes containing 40 g of dust to evaluate surface film formation and antifreeze efficacy. Results showed insufficient coverage and poor antifreezing at volumes below 2.0 L/m², while 2.0 L/m² and 2.4 L/m² achieved complete coverage with superior film quality and antifreeze performance. Balancing cost and effectiveness, 2.0 L/m² was selected for field trials. The detailed dust suppressant application procedure is illustrated in Fig 8.

**Fig 9:**
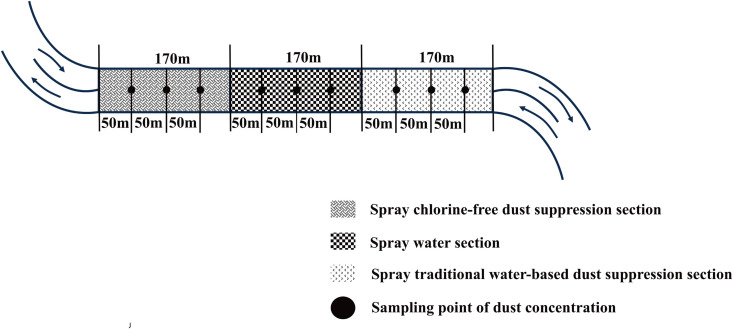
Schematic diagram of the test section plan and dust measurement points.

Fig 9 illustrated the layout of experimental road sections and dust concentration measurement points on surface coal mine roads. Based on site conditions, the truck transport route was divided into three sections, with four dust measurement points established accordingly. These included sections sprayed with chlorine-free dust suppressant (sections 1–2), daily water sections (sections 2–3), and treated with traditional aqueous dust suppressant (sections 3–4). Within each section, three measurement points were set to monitor dust concentration. Each experimental spraying zones covered 6,800 m^2^ (170 m × 40 m), with a fixed rate of 2 L/m^2^ for both water or dust suppressant. The experimental design accounted for branch intersections and road gravel conditions to ensure consistent and uniform spraying across all sections, maintaining an equal spraying volume per unit area.

#### 4.3.2. Data analysis of meteorological conditions and dust concentrations.

1. On-site meteorological data analysis

During the 8 days observation at surface mine site, morning temperatures ranged from −31°C to −17°C, while afternoon varied between −20°C to −11°C. Relative humidity was generally between 50% to 65% in the mornings, and 24% to 43% in the afternoons. Wind speed mostly remained within 5 km/h. Overall, weather conditions were stable, except for isolated abnormal intervals.

1. Spraying dust suppressant road of total dust and respiration dust concentrations.

**Fig 10:**
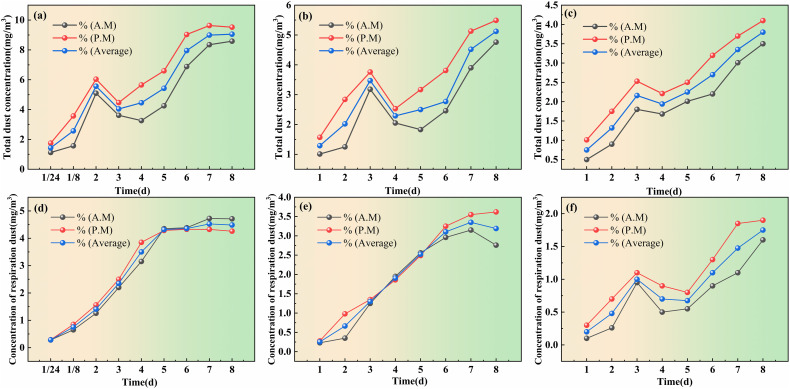
Spraying dust suppressant road total dust and respiration dust concentrations. Total dust concentrations: (a)water, (b) aqueous dust suppressant and (c) chlorine-free dust suppressant; respiration dust concentrations: (d) water,(e) aqueous dust suppressant and (f) chlorine-free dust suppressant.

Fig 10 showed the total dust concentration and respiration dust levels in test section treated with chlorine-free dust suppressant, water and aqueous dust suppressants during field trials. To evaluate dust suppression, water was sprinkled on the sprinkler section, and dust concentration was detected every 20 minutes. According to the national standard, the dust concentration limit for surface mining area is 4 mg/m^3^. After sprinkling, dust concentration ranged between 0.07 and 5.52 mg/m^3^ within 120 minutes. Dust levels remained below the national standard for approximately 110 minutes, indicating temporary suppression effect; however, after 120 minutes, concentrations exceeded the limit and peaked at 10.14 mg/m^3^ at 160 minutes, Therefore, water sprinkling effectively suppressed dust for about 2 hours.

The total dust concentration and concentration of respiration dust at 180 min after water spraying were 1.43 mg/m^3^-1.2 mg/m^3^, respectively, indicating that water sprinkling had a certain dust suppression effect. Both parameters increased rapidly afterward, surpassing 4 mg/m³ and 2.5 mg/m³ by the fourth day (8.35 mg/m³ and 4.3 mg/m³, respectively), highlighting the need for repeated application and higher water volumes.

Comparing aqueous dust suppressant, chlorine-free dust suppressant demonstrated longer efficacy. On the eighth day after application, the total dust concentration in chlorine-free treated scetions peaked at 3.8 mg/m^3^, which below the limit of 4 mg/m^3^ and respirable dust concentration reached 1.75 mg/m^3^ under the limit of 2.5 mg/m^3^. In contrast, sections treated with aqueous suppressants exceeded these limits by the fourth to fifth day, reaching 5.12 mg/m³ of total dust and 3.19 mg/m³ of respirable dust on 8th day. The respirable dust concentration of traditional suppressants surpassed 2.5 mg/m³ by day five, indicating an effective suppression period of approximately four days.

sections treated with aqueous suppressants exceeded these limits by the fourth to fifth day, reaching 5.12 mg/m³ total dust and 3.19 mg/m³ respirable dust on day eight. The respirable dust concentration of traditional suppressants surpassed 2.5 mg/m³ by day five, indicating an effective suppression period of approximately four days.

#### 4.3.3. The consolidation situation of the transport road surface after spraying the dust suppressant.

These results demonstrated the significant effectiveness of the chlorine-free dust suppressant in dust suppression. Despite external interferences during field test, such as airborne dust from discharge area, vehicles activity at branch intersection, and machine damage to the road surface, the dust concentration remained within the limit prescribed by the national standard for 8 consecutive days. In contrast, the section treated with water maintained effective dust suppression for only 2 hours.

**Fig 11:**
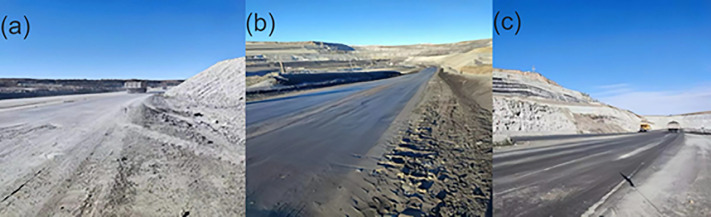
Pavement comparison of pavement sprayed with water (a), chlorine-free dust suppressant (b) and conventional aqueous dust suppressant (c).

As shown in Fig 11, the pavement sprayed with chlorine-free dust suppressant exhibited a distinct solidified layer of about 2–4 cm thick, unlike the pavement sprayed with water or conventional aqueous dust suppressant. This solidification indicated that not only effective dust suppression but also enhanced solidification performance

During the field experiment, ambient temperature in the surface mine fluctuated dramatically between −11°C to −31°C. Despite the extreme conditions, the road surface sprayed with the dust suppressant remained unfrozen and completely moisture, demonstrating strong antifreeze properties.

Overall, the chlorine-free dust suppressant showed excellent dust suppression performance, good environmental stability, and long-lasting effectiveness, making it particularly suitable for the complex conditions of surface mines.

### 4.4. Economic benefit analysis

During winter field trials at the surface mine, spraying water to suppress road dust proved problematic in freezing conditions due to quickly froze, rendering it unsuited for safety standards and on-site operations. Consequently, water spraying was excluded from the dust control strategy.

Previously, the opencast mine exclusively used aqueous dust suppressants. For this trial, we compared the cost-effectiveness of our self-developed chlorine-free suppressant with the traditional aqueous version. A detailed cost analysis showed the aqueous product cost 900 RMB per cubic meter, while chlorine-free formula priced at just 426 RMB per cubic meter. Over six month winter, the estimated dust suppressant usage for mine roads was about 4,500 cubic meters. Our proprietary chlorine-free formula provides substantial cost savings ¥2.13 million less than conventional aqueous systems, a 77.2% cost reduction. This highlights two key advantages: it solves subzero freezing during operations while maintaining performance in winter conditions, and offers remarkable economic efficiency that outperforms traditional methods.

## 5. Conclusion

The optimal monomer materials for surfactant, moisturizer, binder, and antifreeze were determined to be sodium dodecylbenzene sulfonate, glycerol, polyacrylamide complexed with ethylene glycol, potassium acetate, and potassium formate, respectively. The orthogonal test results showed the concentrations of each monomer material under the optimal formulation were 0.25%, 3%, 0.07%, 29%, 4%, and 11%, respectively. This composition resulted in a freezing point of –35.3 °C. The formulation exhibited favorable properties, including good fluidity at low temperatures (viscosity of 12.1 mPa·s at 5 °C and 11.7 mPa·s at 10 °C), superior dust wettability compared to water, minimal corrosiveness, and strong resistance to wind erosion and evaporation.

Field test demonstrated that the suppressant maintained effective dust control for up to 8 days, while water spraying lasted only 2 hours. These results confirmed the chlorine-free antifreeze dust suppressant’s effectiveness and application value for road dust control for coal mines in alpine areas.

The self-developed antifreeze dust suppressant currently has some issues: the field experiment cycle is relatively short, and the existing experimental data are difficult to fully cope with variations in winter temperatures across different years. Additionally, the screening of dust suppressant formulations is time consuming. Further research can introduce molecular simulation methods to explore the intermolecular interaction and simulate its performance under various environmental conditions such as different temperatures and humidity levels, thereby effectively reducing experimental costs.
